# The effect of unilateral and bilateral laparoscopic surgery for endometriosis on Anti-Mullerian Hormone (AMH) level after 3 and 6 months: a systematic review and meta-analysis

**DOI:** 10.1186/s12955-020-01561-3

**Published:** 2020-09-24

**Authors:** Anisodowleh Nankali, Mohsen Kazeminia, Parnian Kord Jamshidi, Shamarina Shohaimi, Nader Salari, Masoud Mohammadi, Amin Hosseinian-Far

**Affiliations:** 1 School of Medicine, Department of Obstetrics and Gynecology, Kermanshah University of Medical Sciences, Kermanshah, Iran; 2 Department of Nursing, School of Nursing and Midwifery, Kermanshah University of Medical Sciences, Kermanshah, Iran; 3 Department of Biology, Faculty of Science, University Putra Malaysia, Serdang, Selangor Malaysia; 4 Department of Biostatistics, School of Health, Kermanshah University of Medical Sciences, Kermanshah, Iran; 5 Department of Business Systems & Operations, University of Northampton, Northampton, UK

**Keywords:** Laparoscopy, Unilateral, Bilateral, Endometriosis, AMH, Meta-analysis

## Abstract

**Background:**

Endometriosis is one of the most common causes of infertility. The causes of the disease and its definitive treatments are still unclear. Moreover, Anti-Mullerian Hormone (AMH) is a glycoprotein dimer that is a member of the transient growth factors family. This research work aimed to identify the effect of unilateral and bilateral laparoscopic surgery for endometriosis on AMH levels after 3 months, and 6 months, using meta-analysis.

**Methods:**

In this study, the articles published in national and international databases of SID, MagIran, IranMedex, IranDoc, Cochrane, Embase, Science Direct, Scopus, PubMed, and Web of Science (ISI) were searched to find electronically published studies between 2010 and 2019. The heterogeneous index between studies was determined using the I^2^ index.

**Results:**

In this meta-analysis and systematic review, 19 articles were eligible for inclusion in the study. The standardized mean difference was obtained in examining of unilateral laparoscopic surgery for endometriosis (before intervention 2.8 ± 0.11, and after 3 months 2.05 ± 0.13; and before intervention 3.1 ± 0.46 and after 6 months 2.08 ± 0.31), and in examining bilateral laparoscopic surgery for endometriosis examination (before intervention 2.0 ± 08.08, and after 3 months 1.1 ± 0.1; and before intervention 2.9 ± 0.23 and after 6 months 1.4 ± 0.19).

**Conclusion:**

The results of this study demonstrate that unilateral and bilateral laparoscopic surgery for endometriosis is effective on AMH levels, and the level decreases in both comparisons.

## Background

Endometriosis refers to the implantation of endometrium tissue, which includes stroma and epithelial tissues outside the uterus. Endometriosis is one of the most common causes of infertility and implantation failure [1]. The study of Kresch et al. showed that among 850 patients who referred to a clinic due to chronic pain (for more than 6 months) and were under laparoscopy, 92% had endometriosis or adhesion [2].

Anti-Mullerian Hormone (AMH) is a glycoprotein dimer that is a member of the transient growth factors family. AMH hormone belongs to the transforming growth factor-b family and is produced by the granulosa cells of primary to small antral follicles [3]. Serum AMH concentration correlates with the number of small follicles, and is impacted by the ovarian reserve rate [4].

During follicle growth, AMH expression is decreased once the follicle reaches a certain size (8 mm), resulting in an increased sensitivity of the follicle to circulating FSH. This reduces AMH level, which in turn provides an environment for follicle growth until ovulation [5].

Various surgical treatments have also been suggested for endometriosis, and significant differences have been reported among the therapeutic results of these methods. However, the preference of most surgeons and patients have recently shifted from open surgeries toward the laparoscopic treatments since laparoscopy is considered as a faster and a less aggressive method. Laparoscopic treatments include endometrioma aspiration, laparoscopic cystectomy, cyst drainage, catheterization with catheter or laser, and even more radical treatments such as removal of a part or the whole ovary, and sometimes with uterine appendages [6]. Moreover, by comparing the possible complications of therapeutic methods with each other, the available evidence indicates that laparoscopic methods are superior. Possible complications in laparoscopic treatments include damage to other organs caused by the laparoscopic devices, a reduced level of AMH, wound infection, bleeding, postoperative morbidity or long-term hospitalization, ileus, deep vein thrombosis, and/or other medical complications [7].

There have been several preliminary pieces of research studying the effect of unilateral and bilateral laparoscopic surgery for endometriosis on AMH level after 3 and 6 months. However, there are contradictions between the results of these studies. One of the applications of meta-analysis studies is to respond to these assumptions and resolve contradictions. Therefore, the aim of this study was to identify the effect of unilateral and bilateral laparoscopic surgery for endometriosis on AMH level after 3 and 6 months using meta-analysis.

## Methods

### Method of searching articles

In this study, the Persian databases of SID, MagIran, IranMedex and IranDoc, and the international databases of Cochrane, Embase, Science Direct, Scopus, PubMed and Web of Science (ISI) were searched without a lower time-limit and until December 2019, with a view to find related articles and reports. The list of references within the identified above sources were manually evaluated to find other possible studies. The keywords used to search references were selected from the Medical Subject Headings (MeSH) thesaurus. Laparoscopic, Unilateral, Bilateral, Anti-Mullerian hormone, AMH, and endometriosis were the selected search keywords. The studies were assessed according to the four-step PRISMA 2009 process, which entails the phases of: identification, screening, eligibility assessment, and finally, including the articles in the meta-analysis.

### Inclusion criteria

Articles with the following characteristics were selected for the meta-analysis: 1) Original research articles, 2) Clinical Trial Studies, 3) Articles with their full-text being available, and 4) Studies that examined the relationship between unilateral and bilateral laparoscopy for endometriosis and AMH.

### Exclusion criteria

The selected studies were examined in more details. Review papers, and studies where their sample were not selected from patients with endometriosis, as well as studies reusing previous data, were excluded from the meta-analysis. Finally, 27 studies were entered into the third stage, i.e. quality evaluation.

### Quality evaluation

In order to evaluate the quality of articles, the CONSORT checklist was used. The checklist has a number of scales that include: study plot, background, literature review, place and time of study, outcome, inclusion criteria, sample size, and statistical analysis. The maximum score that could be obtained during the quality evaluation and using this checklist is 40. Studies with a score of less than 19 were considered as low-quality articles, and therefore were excluded from the study [8]. In this research, 19 articles that were assessed as medium or high-quality articles were entered in the final systematic review and meta-analysis phase, and 8 articles which were scored as low-quality studies were excluded.

### Data extraction

The data from the final selection of sources were extracted using a different pre-prepared checklist. The checklist includes a number of fields such as the article title, first author’s name, year of publication, place of study, sample size of unilateral and bilateral laparoscopic surgery for endometriosis intervention groups, mean sample before intervention, mean sample after 3 and 6 months, the standard deviation of the sample before intervention, and the standard deviation of the sample after 3 and 6 months.

### Statistical analysis

Since the focus of the research was in relation to the effect of unilateral and bilateral laparoscopic surgery for endometriosis on AMH level after 3 and 6 months, frequency and percentage, as well as standardized mean difference in examining unilateral and bilateral laparoscopic surgery for endometriosis before and after intervention in each study, were used to combine the reported results of the collected studies. The I^2^ index was initially used to investigate the homogeneity among the studies; and since the reported results were found to be heterogeneous, the random effects model was used to combine the findings and perform the meta-analysis. When the I^2^ index was less than 25%, it was considered as low heterogeneity, between 25 and 75% as moderate heterogeneity and more than 75% as high heterogeneity. *P*-value less than 0.05 was considered statistically significant. The Egger’s test was also used to investigate the publication bias. The data were analyzed using the Comprehensive Meta-Analysis software (Biostat, Englewood, NJ, USA Version 3).

The standardized mean difference index and 95% confidence interval in every study, as well as the final estimation of the index obtained from the combination of studies, have been illustrated in the Forrest plot. In this plot, the weight of every study has been shown in the final combined value, and the size of every square is proportional to the weight of that study in the meta-analysis.

## Results

**Fig. 1:**
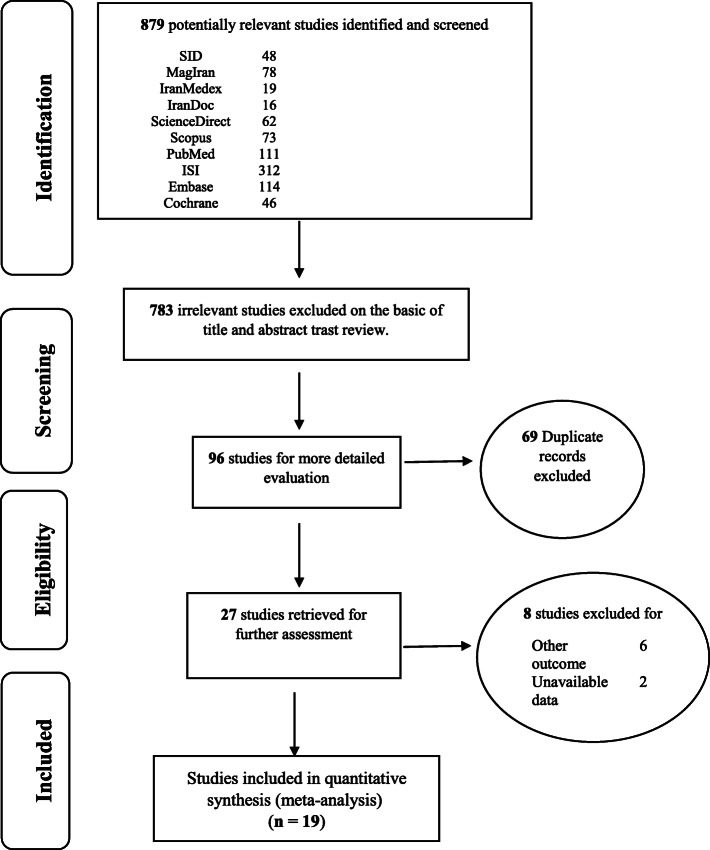
Flow diagram of study selection

In this research work, all studies focusing on the comparison of unilateral and bilateral laparoscopic surgery for endometriosis on AMH level after 3 and 6 months were systematically examined without time limitations and according to the PRISMA guidelines. In the initial search, 879 articles were identified. This number was reduced to 19 for the final analysis, and includes articles that were published between 2010 and December 2019 (Fig. 1).

The characteristics of studies entered into the systematic review (Tables 1 and 2).

**Table 1:** Characteristics of studies performed on meta-analysis in unilateral endometrial laparoscopic group

Author, year, Reference	Place of study	sample size	Mean ± SD of Before	Mean ± SD of 3 months	Mean ± SD of 6 months	Quality
Suksompong, 2012, [9]	Thailand	28	2.14	1.45	–	High
Ergun, 2011, [10]	Turkey	50	2.03 ± 0.41	1.95 ± 0.62	–	High
Ergun, 2015, [11]	Turkey	38	3.15 ± 2.86	2.10 ± 1.82	–	High
Hwu, 2011, [12]	Taiwan	147	2.48	1.33	–	High
Chang, 2010, [13]	Korea	20	2.78	1.8	–	Medium
Mostaejeran, 2015, [14]	Iran	33	3.08	1.75	–	Medium
Adnyana, 2018, [15]	Indonesia	25	2.17 ± 1.24	1.79 ± 0.97	–	High
Chun, 2015, [16]	Korea	26	4.97 ± 2.66	3.59 ± 1.83	–	High
Salihoğlu, 2016, [17]	Canada	34	3.50 ± 2.70	2.70 ± 1.40	–	High
Alborzi, 2014, [6]	Iran	193	4.31 ± 3.82	2.53 ± 2.92	–	High
El-Dorf-1, 2015, [18]	Egypt	120	3.10 ± 0.31	2.50 ± 0.11	2.20 ± 0.27	High
El-Dorf-2, 2015, [18]	Egypt	80	2.40 ± 0.22	2.00 ± 0.23	1.90 ± 0.21	High
Nappi, 2016, [19]	Italy	45	3.01 ± 0.78	–	2.76 ± 0.8	High
Saito, 2018, [20]	Japan	32	4.40 ± 2.55	–	1.2 ± 1.3	High
Marshall, 2019, [21]	Sudan	20	3.22	–	1.82	Medium
Shao, 2016, [22]	China	36	5.02 ± 3.05	–	4.43 ± 2.13	High
Celik, 2012, [23]	Turkey	39	1.05	–	0.6	High

**Table 2:** Characteristics of studies performed on meta-analysis in bilateral endometrial laparoscopic group

Author, year, Reference	Place of study	sample size	Mean ± SD of Before	Mean ± SD of 3 months	Mean ± SD of 6 months	Quality
Suksompong, 2012, [9]	Thailand	15	2.11	0.74	–	High
Ergun, 2015, [11]	Turkey	12	1.18 ± 1.07	1.00 ± 1.01	–	High
Hwu, 2011, [12]	Taiwan	147	1.7	1.03	–	High
Chang, 2010, [13]	Korea	20	2.18	0.95	–	Medium
Adnyana, 2018, [15]	Indonesia	35	2.07 ± 1.18	1.44 ± 0.87	–	High
Sumapraja, 2011, [24]	Indonesia	22	1.64	0.6	–	High
Salihoğlu, 2016, [17]	Canada	34	2.60 ± 2.30	2.20 ± 1.9	–	High
Alborzi, 2014, [6]	Iran	193	2.60 ± 1.98	1.07 ± 0.97	–	High
Tanprasertkul, 2014, [25]	Thailand	39	2.01	1.60	1.68	High
El-Dorf-2, 2015, [18]	Egypt	50	2.90 ± 0.40	1.70 ± 0.30	1.6 ± 0.12	High
El-Dorf-2, 2015, [18]	Egypt	80	1.90 ± 0.24	1.30 ± 0.10	0.97 ± 0.11	High
Saito, 2018, [20]	Japan	37	3.1 ± 1.7	–	0.8 ± 0.7	High
Marshall, 2019, [21]	Sudan	39	3.19	–	0.88	Medium
Shao, 2016, [22]	China	36	4.68 ± 2.87	–	3.05 ± 1.99	High
Zaitoun, 2013, [26]	Egypt	61	4.50 ± 0.80	–	2.40 ± 0.50	Medium
Celik, 2012, [23]	Turkey	39	2.20	–	0.55	High

The standardized mean difference indices in the articles were used to examine the effects of the reported results in the studies. In the articles where standard deviation ± mean was reported, the standardized mean difference index was used in the meta-analysis. The results of the meta-analysis showed heterogeneity in studies examining unilateral laparoscopic surgery for endometriosis after 3 and 6 months (I^2^ = 99.9) and in research works examining bilateral laparoscopic surgery for endometriosis after 3 and 6 months (I^2^ = 99.8). Considering this, the random effects model was adopted to enable the amalgamation of the reported results.

The Egger’s test was used to investigate the presence of publication bias in the studies. According to the Egger’s test results, there was no publication bias in studies a) examining unilateral laparoscopic surgery for endometriosis after 3 and 6 months (*P* = 0.244), b) examining bilateral laparoscopic surgery for endometriosis after 3 and 6 months (*P* = 0.891).

**Fig. 2:**
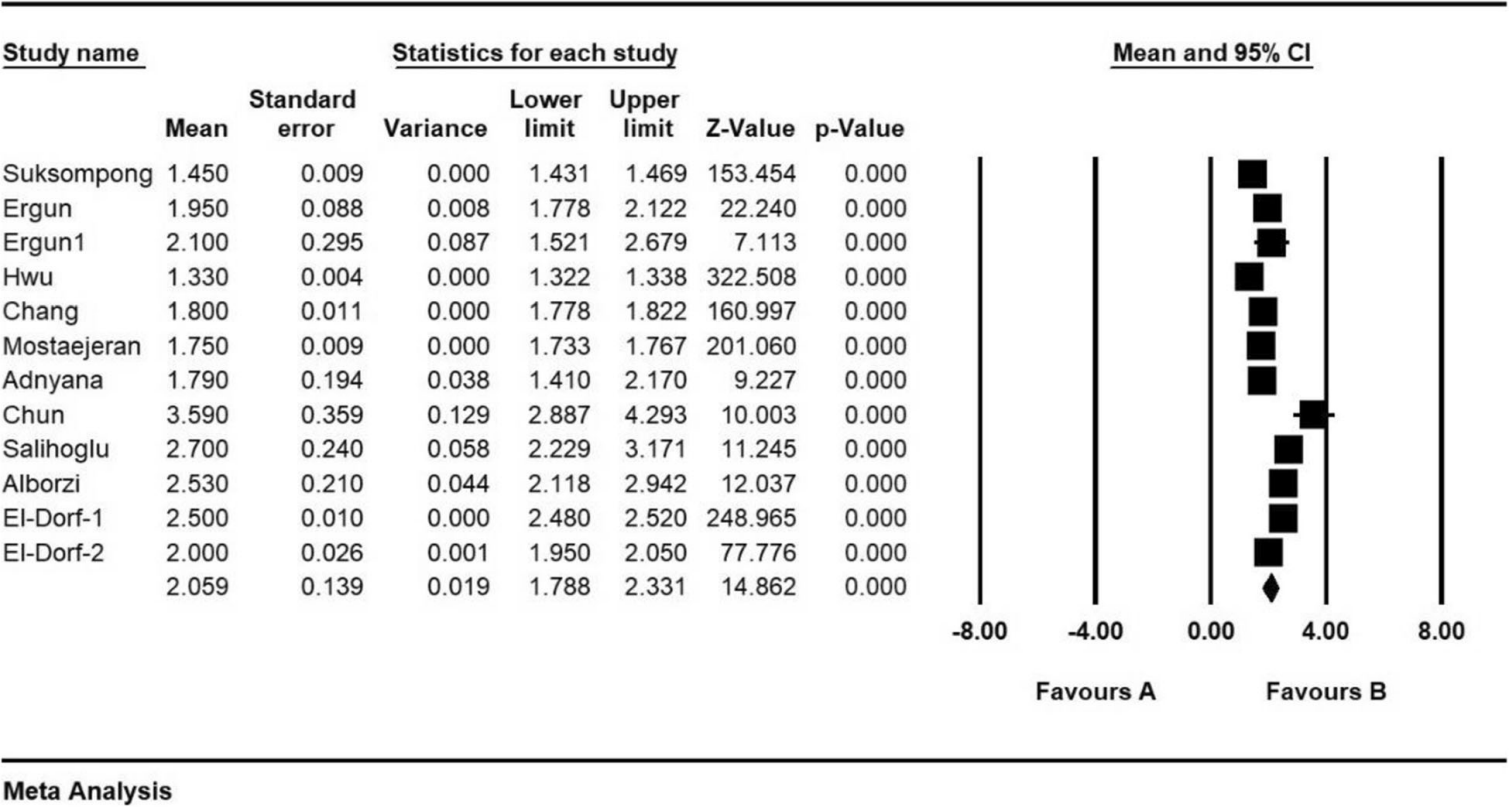
Accumulation plot obtained from studies entered in Meta-analysis analysis using standardized mean difference index of AMH changes with unilateral laparoscopic surgery of endometriosis after 3 months

**Fig. 3:**
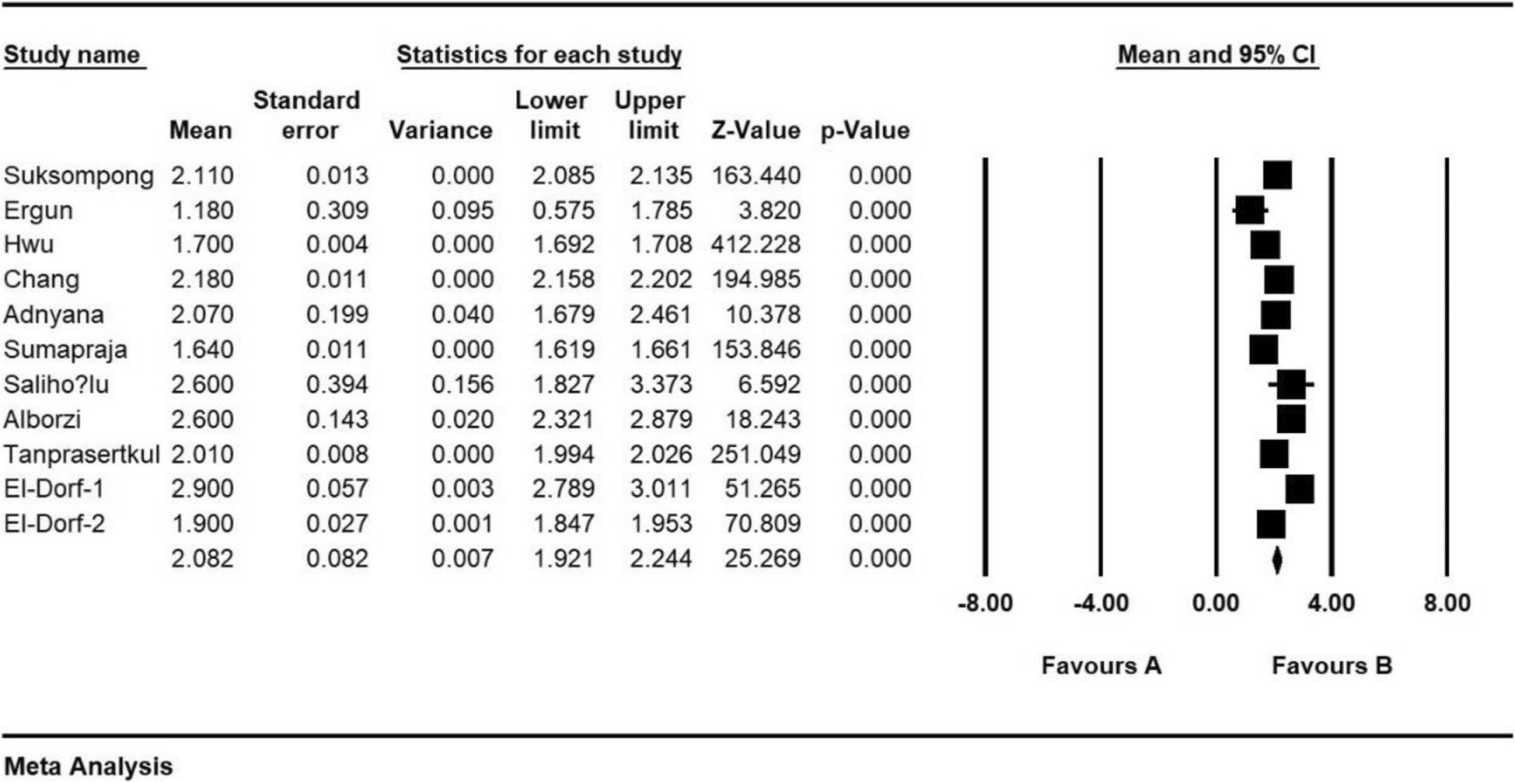
Accumulation plot obtained from studies entered in Meta-analysis analysis using standardized mean difference index of AMH changes with unilateral laparoscopic surgery of endometriosis after 6 months

**Fig. 4:**
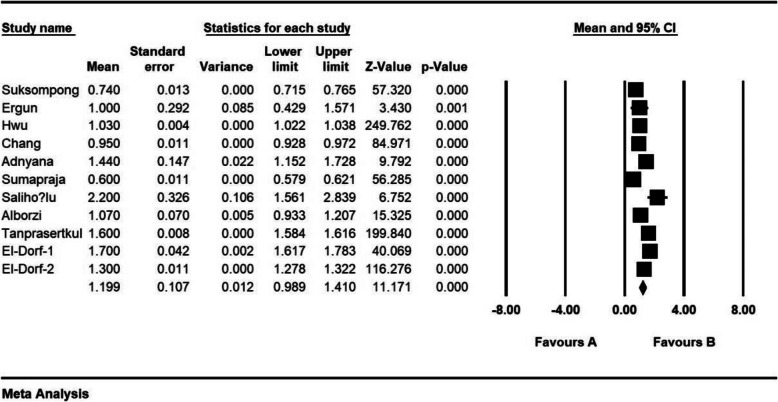
Accumulation plot obtained from studies entered in Meta-analysis analysis using standardized mean difference index of AMH changes with bilateral laparoscopic surgery of endometriosis after 3 months

**Fig. 5:**
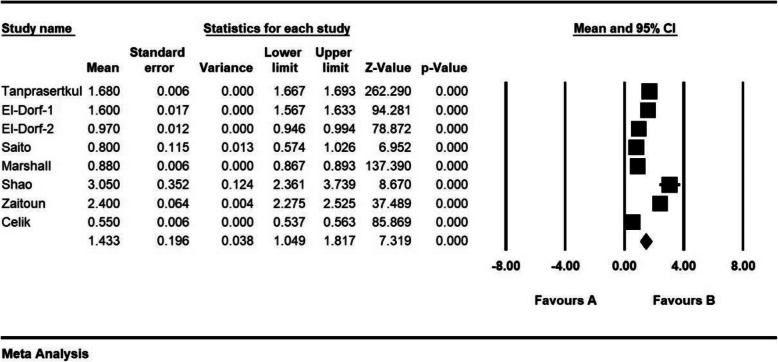
Accumulation plot obtained from studies entered in Meta-analysis analysis using standardized mean difference index of AMH changes with bilateral laparoscopic surgery of endometriosis after 6 months

**Fig. 6:**
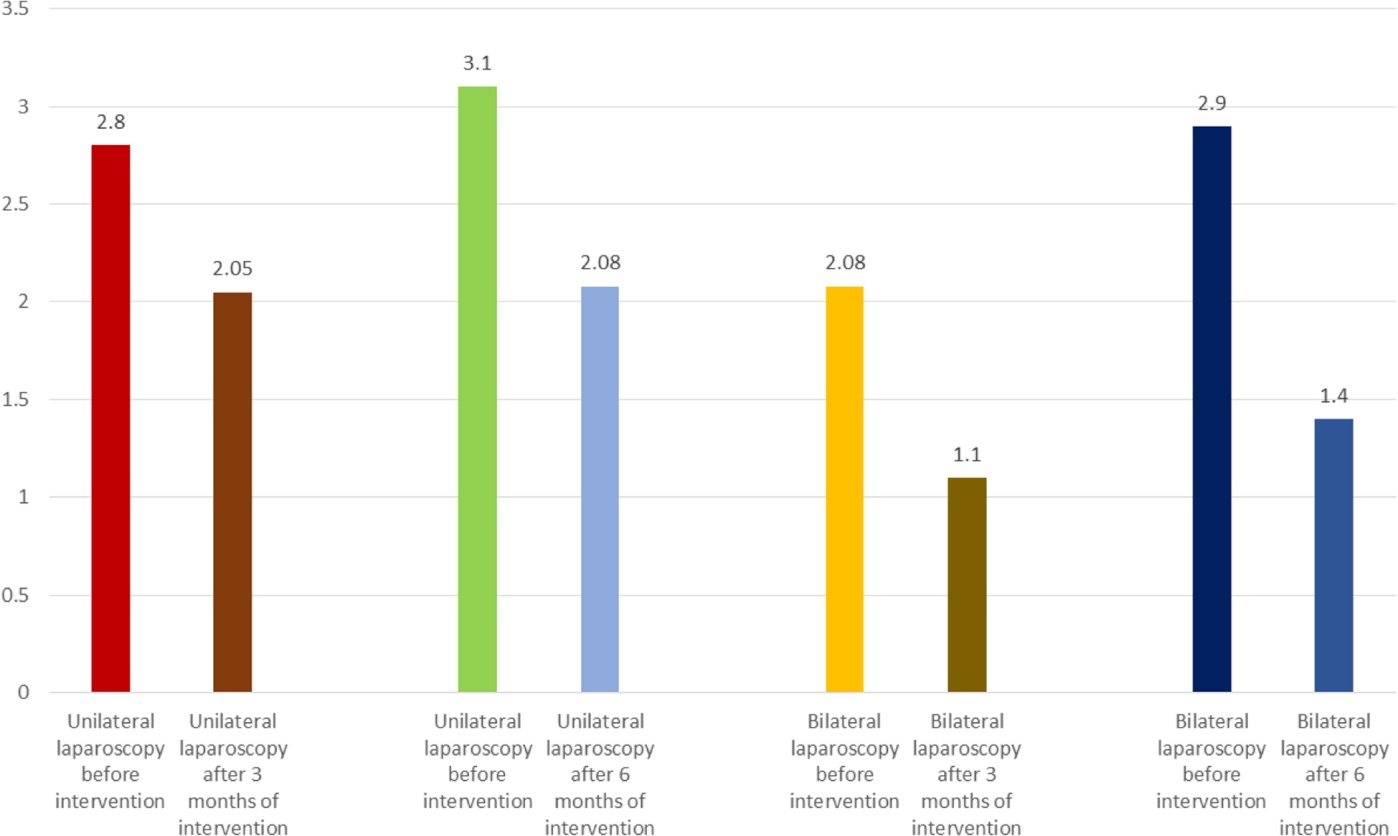
Overall Standardized mean difference in bilateral and unilateral laparoscopic surgery of endometriosis before the intervention and after 3 and 6 months

The standardized mean difference was obtained in examining unilateral laparoscopic surgery for endometriosis (before intervention 2.8 ± 0.11 and after 3 months 2.05 ± 0.13; and before intervention 3.1 ± 0.46 and after 6 months 2.08 ± 0.31), and in examining bilateral laparoscopic surgery for endometriosis (before intervention 2.08 ± 0.08 and after 3 months 1.1 ± 0.1; before intervention 2.9 ± 0.23 and after 6 months 1.4 ± 0.19), which indicates unilateral and bilateral laparoscopic surgery for endometriosis is effective on AMH level. The AMH level is reduced in both comparisons, and the effect of bilateral laparoscopic surgery for endometriosis on AMH level was more than for unilateral laparoscopic surgery for endometriosis. Moreover, this reduction increases after 6 months. (Figs. 2, 3, 4, 5, 6).

## Discussion

The aim of this study was to investigate the effect of unilateral and bilateral laparoscopic surgery for endometriosis treatment on the reduction of AMH level after 3 and 6 months, using meta-analysis, in which the standardized mean difference was obtained in examining unilateral laparoscopic surgery for endometriosis, and in examining bilateral laparoscopic surgery for endometriosis.

In a meta-analysis conducted by Amer et al. (2017) using 7 articles, the level of AMH after laparoscopy was reported as 2.13 ng/ml [27]. This difference between this and our findings may be due to the fact that in our study the articles related to unilateral and bilateral laparoscopic surgery for endometriosis have been studied separately.

In vitro fertilization (IVF) is a complex series of procedures used to help with fertility or prevent genetic problems and assist with the conception of a child, During IVF, mature eggs are collected from ovaries and fertilized by sperm in a lab. Then the fertilized egg (embryo) or eggs (embryos) are transferred to a uterus [27–29].

In a meta-analysis which was performed on women with endometrium under IVF between 1985 and 2007, 20 pieces of research were investigated; the meta-analysis demonstrated that the clinical pregnancy rate was not different between the treated and untreated groups [29].

Cystectomy is a common treatment among endometriosis surgical treatments. In laparoscopic cystectomy method, which is still considered the preferred therapeutic method, the inner layer of the cyst is separated, and removed from the ovarian tissue by two forceps, which may affect ovarian reserve [6].

According to a meta-analysis conducted by Somigliana et al. (2012), changes in AMH serum level after endometrial ablation, affect the damages caused to the ovarian reserve following the surgery [30]. Chang et al. (2010) [13] in a different study in China on 60 infertile women measured levels of AMH, inhibin B, FSH, LH, and estradiol on third day of the menstrual cycle and found that there was a significant difference between AMH levels on the third day of the menstrual cycle in fertile and infertile women, and concluded that AMH is correlated better with the number of recovered oocytes than with age, FSH and inhibin B. Women whose AMH levels were within the range of 4.4 ± 2.2 had a better response than women whose AMH serum levels were 0.7 ± 0.8 (*P* < 0.01) [13].

The study of Visser et al. (2006) in the Netherlands showed that AMH is a quantitative marker of ovarian reserve as well as ovarian dysfunction. Unlike FSH, AMH can be measured on any day of the cycle. The level of AMH decreases with performing laparoscopy, and it can be predicted that the ovarian efficacy is reduced by decreasing the AMH value [31].

Lekamge et al. (2007) in a study in Australia measured the baseline concentration of AMH, FSH, and antral follicle count from 126 women undergoing IVF treatment, and concluded that in patients with low AMH, lower oocytes number were formed than average persons (*P* < 0.01). The fertility rate was also lower in the study group (*P* < 0.05) and fewer embryos were formed (*P* < 0.05) [32].

According to this systematic review and meta-analysis study, the effect of bilateral laparoscopic surgery for endometriosis on AMH level was more than for unilateral laparoscopic surgery for endometriosis. This study demonstrates that the level of AMH is visibly reduced after endometriosis laparoscopy. This reduction of AMH levels does not stop after 6 months, but decreases even further. Therefore, gynaecologists should not overlook the decrease of AMH levels after unilateral and bilateral laparoscopic endometrial surgeries, since the decreased ovarian reserve may result in infertility, or premature menopause in future.

Our study had a few limitations; the heterogeneity of the patient’s population, in terms of age and extent of endometriosis, may have an effect on the scientific validity of the reported results. In some articles, a follow-up was performed shortly after the intervention (3 months), however, the number of articles studying 6 months after the intervention was limited. Therefore, it is suggested to conduct another meta-analysis for examining AMH levels after 9 months and 1 year.

## Conclusion

The results of this study indicate that unilateral and bilateral laparoscopic surgery for endometriosis is effective on AMH level and the level decreases for both comparisons. The effect of bilateral laparoscopic surgery for endometriosis on AMH level reduction is more than unilateral laparoscopic surgery for endometriosis; moreover this reduction intensifies after 6 months. Therefore, the findings in this research work can be beneficial for health policy makers and professionals in this field.

## Data Availability

Datasets are available through the corresponding author upon reasonable request.

## Abbreviations

AMH: Anti-Müllerian Hormone
FSH: Follicle-Stimulating Hormone
CONSORT: Consolidated Standards of Reporting Trials
PRISMA: Preferred Reporting Items for Systematic Reviews and Meta-Analysis
